# Expanding the Scope of Administrative Health Records Through Advanced Statistical Methods

**DOI:** 10.1093/geroni/igab046.1062

**Published:** 2021-12-17

**Authors:** Igor Akushevich, Carl V Hill, Konstantin Arbeev

## Abstract

The objective of the Symposium is to expand familiarity of the application of advanced methods of modern statistical modeling and data management, to administrative health data by combining methodological innovations with practical hands-on demonstrations. Topics will cover a range of methodological and substantive topics including: i) decomposition and partitioning approaches in analysis of disparities and time trends in AD/ADRD; ii) new artificial intelligence technologies that allow us to enrich electronic health record datasets with self-report scores in geriatrics; iii) using administrative data to model adherence to disease management and health-related behavior; iv) the use of longitudinal extension of the average attributable fraction to study health disparities and multimorbidity; and v) the geographic and racial disparities in total and remaining life expectancies after diagnoses of AD/ADRD and other chronic conditions. The increasing availability of large-scale datasets based on electronic health records and administrative claims records provide an unprecedented opportunity for obtaining nationally representative results based on individual-level measures that reflect the real care-related and epidemiological processes. This makes the reduction of barriers to entry to the use of such data of vital importance to the community of geriatrics and health researchers.

